# Parameters of glycemic variability in continuous glucose monitoring as predictors of diabetes: a prospective evaluation in a non-diabetic general population

**DOI:** 10.1515/almed-2025-0011

**Published:** 2025-03-07

**Authors:** Javier Rodríguez García, Felix Camiña Darriba, Juan B. Ortolá Devesa, Santiago Rodríguez-Segade Villamarín, Andrea Valle Rodríguez

**Affiliations:** Laboratorio de Bioquímica Clínica del Complejo Hospitalario Universitario de Santiago de Compostela, Santiago de Compostela, Spain; Departamento de Bioquímica y Biología Molecular, Universidad de Santiago de Compostela, Santiago de Compostela, Spain

**Keywords:** continuous glucose monitoring, diabetes, glycemic variability, HbA_1c_, mean amplitude of glucose excursions, standard deviation

## Abstract

**Objectives:**

To prospectively examine the ability of some glycemic variability metrics from continuous glucose monitoring (CGM) to predict the development of diabetes in a non-diabetic population.

**Methods:**

A total of 497 non-diabetic patients from the AEGIS study were included. Participants used a CGM system (iPro2^®^) over a six-day period. The following parameters were analyzed: standard deviation (SD), coefficient of variation (CV) and mean amplitude of glucose excursion (MAGE). Six-years follow-up was performed. ROC curves were constructed to determine the predictive value of glycemic variability metrics. Sensitivity and specificity were calculated.

**Results:**

Of the 497 participants, 16 women (4.9 %) and 9 men (5.2 %) developed diabetes. Initial HbA_1c_ and fasting glucose levels were significantly higher in the participants who ultimately developed diabetes. Glycemic variability metrics were also significantly higher in these subjects (SD: 18 vs. 13 mg/dL; CV: 17 vs. 14 %; MAGE: 36 vs. 27 mg/dL; p<0.001 in all cases). SD showed the highest AUC (0.81), with a sensitivity of 80 % and a specificity of 72 % for a cut-off of 14.9 mg/dL. AUCs were higher in men for all metrics.

**Conclusions:**

The metrics obtained by MCG, especially SD, are effective predictors of progression to type 2 diabetes in a non-diabetic population. These findings suggest that glycemic variability is useful for the early identification of subjects at a higher risk of developing diabetes.

## Introduction

Continuous glucose monitoring (CGM) systems are small devices fitting a subcutaneous sensor that provides detailed information about glucose variations. This technology allows to asses the magnitude and duration of glucose variations more accurately than conventional methods [1], 2]. Although CGM is very useful for controlling and monitoring diabetic patients, its implementation presents some challenges for health professionals, who may find it difficult to manage and clinically use the data obtained. In the recent years, CGM measures, including glycemic variability and time in range, have been integrated into routine clinical practice [3]. In 2019, a panel of experts in CGM technologies (clinicians, researchers and patients with diabetes) published a set of consensus recommendations to standardize an appropriate use of time-in-range metrics in clinical practice. A more recent review established time in range as the gold-standard measure [4], 5]. Additionally, time in range is widely accepted as a predictor of complications of diabetes [6].

A range of studies performed in diabetic patients, treated or not with insulin demonstrate the benefits of CGM, in loss of weight, improvement of dietary habits and/or increase in physical activity [7], [8], [9], [10], [11], [12], [13]. However, CGM is only indicated for diabetic patients.

The methods currently available for establishing diagnosis of prediabetes provide a snapshot of the glycemic status of a subject. However, these methods can be misleading and/or ineffective in timely detecting, controlling and managing dysglycemia. Moreover, HbA_1c_ determination can be falsely elevated or reduced in subjects with hemoglobinopathies, chronic kidney disease, anemia and other interfering factors [14], [15], [16]. Additionally, test results may be influenced by ethnic differences in glycosylation rates and during pregnancy [17], [18], [19], [20]. HbA_1c_ values only account for mean glucose levels over a period of 2–3 months. This prevents the identification of specific disorders or behaviours of the patient that contribute to dysglycemia, which is essential for an effective glycemic control.

The use of CGM in subjects at risk (for example, overweight/obesity, familial history of T2D) may overcome the limitations of the methods currently available. CGM provides significant information in a format that enables clinicians and patients to easily identify glycemic patterns and within-day and between-day glucose variations that may indicate the presence and/or severity of dysglycemia. Our previous results in non-diabetic patients reveal that subjects with normal HbA_1c_ and fasting glucose (FG) levels, that spend a high percentage of time in glucose levels in prediabetes and/or diabetes range, also exhibit higher glucose variability (GV) as compared to subjects with normal glucose levels. Glucose variability in the former is similar to those of subjects identified as prediabetic according to conventional criteria [21].

The primary objective of this study was to prospectively assess whether the use of three glycemic variability metrics initially determined by CGM in a large representative sample of a non-diabetic population are associated with a higher risk for progression to type 2 diabetes. These metrics included coefficient of variation (CV), standard deviation (SD) and mean amplitude of glucose excursions (MAGE). MAGE measures glycemic variability during CGM by measuring the mean amplitude of glucose fluctuations exceeding a standard deviation, being associated with a higher risk for progression to type 2 diabetes.

## Materials and methods

### Participants and study design

The participants in this study were extracted from the 1,516 cohort of the *Estudio de Glicación e Inflamación at A Estrada* (AEGIS; NCT01796184 trial at www.clinicaltrials.gov) [22], initiated in 2012. AEGIS is a prospective, population-based, epidemiological study assessing the association between different dysglycemia tests and the risk for progression to diabetes and cardiovascular disease.

Of the 1,516 participants, 1,065 complied with the basic requirements for CGM (capacity to comply with the protocol, refraining from eating out, and absence of allergy to adhesives or any other disease that may influence the data collected by CGM). Of the 1,065 subjects, 622 agreed to take part in the study and underwent CGM over a six-day period. Of the 622 patients, 497 were included in the statistical analysis, as they met the following additional criteria [1]: being clinically stable, not having any acute disease or history of diathesis or chronic kidney or liver disease [2]; fasting glucose <126 mg/dL and HbA_1c_ < 6.5 % [48 mmol/mol] when they had the CGM sensor placed and a week after its removal [3] not using any medication that may influence glucose metabolism over the CGM period [4]; not being pregnant [5]; subjects with incomplete CGM readings were excluded (<2 entire days). Diagnosis of diabetes was established according to the American Association of Diabetes criteria [23].

### Continous glucose monitoring (CGM)

A detailed analysis of CGM has been provided elsewhere [21]. Briefly, on day 0 the selected participants had the CGM device (iPro2^®^ de Medtronic Minimed, Northridge, CA) placed by a trained nurse after overnight fasting. One hour later, blood was drawn to determine fasting glucose and HbA_1c_ levels. Instructions to use and calibrate CGM device were provided to the participants, who wore the device for seven consecutive days without changing their dietary or physical activity habits. To calibrate the CGM device, participants learned to use a glucose test device that provides similar plasma glucose values (OneTouch Verio Flex^®^ de LifeScan, Milpitas, CA). Participants were instructed to calibrate the MCG device at least three times a day (before meals and at bedtime). They were also asked to keep a simple record of their physical activity, dietary intake and hours of sleep. The CGM device was removed on day 7 after overnight fasting. Blood was drawn to determine fasting glucose and HbA_1c_.

All 24-h CGM readings were excluded if (a) the absolute difference between mean relative capillary blood readings that day and the corresponding CGM values exceeded 18 %; and (b) the 24-h MCG readings was incomplete (288 readings between 12 a.m. and 12 p.m.). Participants with less than two complete 24-h readings were excluded from the study. Mean 24-h glycemia (24 h-GM) was estimated as the mean of the 288 readings in a day; 24-h SD as the standard deviation of these 288 readings; 24-CV as (24-h SD)/(24 h-GM), and MAGE (mean amplitude of glycemic excursions) as described by Hill et al. [24].

### Biochemical analyses

Glucose was determined in serum samples of participants in fasting conditions by the glucose oxidase-peroxidase method. Triglycerides, HDL, LDL, total cholesterol and markers of liver and kidney function were determined by enzymatic methods in an autoanalyzer (Advia 2400 Siemens Healthcare Diagnostics, Barcelona, Spain). Capillary glycemia was determined by using glucometers (LifeSpan OneTouch^®^ Verio^®^ Pro). HbA_1c_ was measured by high-resolution liquid chromatography in a Menarini Diagnostics HA-8160 analyzer. All HbA_1c_ values were converted into values aligned with the Diabetes Control and Complications Trial (DCCT) [25]. According to ADA criteria [23], normal glucose values were defined as glucose levels <100 mg/dL and HbA_1c_ levels < 5.7 %. All laboratory tests were performed on the same day as the blood drawn at the Clinical Biochemistry Laboratory of Santiago de Compostela Hospital, Spain.

### Statistical analysis

All variables showed a normal distribution. Continuous variables are presented as means ± SD or as medians with interquartile ranges between brackets. Descriptive statistics were used for the total sample. Statistically significant differences were established by Student’s t-test for parametric variables, or by Mann–Whitney U test for non-parametric variables. Matched correlations between variables were calculated by Pearson r or Spearman’s rho. Two-tailed p-value with an α level of significance was established at 0.05. All statistical analyses were performed with SPSS v27 Chicago, IL).

### Ethics statement

This study was approved by the Ethics Committee for Clinical Research of Galicia, Spain (CEIC# 2012-025 y CEIC# 2016-240). Written informed consent was obtained from all participants, in accordance with the Declaration of Helsinki.

## Results

**Figure 1:**
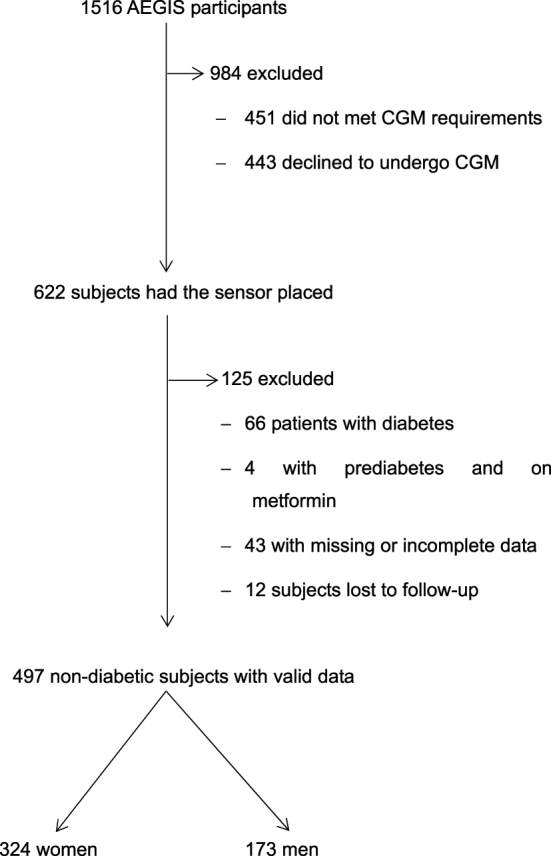
Patient selection.

In total, 497 of the 622 participants of this study met the inclusion criteria (Figure 1). Forty-three subjects were excluded due to missing or inaccurate data or due to participant’s difficulty in operating the device. Subsequently, another 70 subjects were excluded, of which 66 had a diagnosis of diabetes and 4 had received metformin for prediabetes. Twelve patients were lost to follow-up (58 % males). The latter were younger (37 ± 18 years) and had a BMI of 26.4 ± 4.3 kg/m^2^. None had metabolic syndrome. Fasting glucose and HbA_1c_ were 88 ± 10 mg/dL and 5.3 ± 0.3 %, respectively.

Participants provided a total of 2,347 entire days on CGM: 80 % of participants provided 5 days; 14.3 % provided 4 days; 3.4 % provided 3 days, and 2.3 % provided 2 days. Of the 497 participants, 324 (65.2 %) were women and 173 (34.8 %) were men. As compared to the female group (Table 1), men exhibited significantly higher BMI, systolic and diastolic pressure, triglycerides, LDL-cholesterol and fasting glucose values (p<0.05), and significantly lower levels of HDL-cholesterol (p<0.001). No statistically significant differences were observed between men and women in baseline glycemic control values (HbA_1c_), glycemic variability values obtained from the CGM (SD, CV and MAGE), or mean glycemia recorded by the sensor.

**Table 1:** Baseline characteristics of participants.

	Total	Men	Women	p-Value^a^
Number of patients	497	173	324	
Age, years	46.6 ± 13.9	46.1 ± 14.1	46.9 ± 13.8	0.537
Active smoking	112 (22.5)	47 (27.2)	65 (20.1)	0.071
BMI, kg/m^2^	27.8 ± 5.1	28.8 ± 5.0	27.2 ± 5.0	0.01
Obesity (BMI ≥30 kg/m^2^)	153 (30.8)	67 (38.7)	86 (26.5)	0.005
Systolic pressure, mmHg	125 ± 15	130 ± 13	123 ± 15	<0.001
Diastolic pressure, mmHg	77 ± 8	80 ± 8	75 ± 8	<0.001
eGFR, mL/min/1,73m^2^	112.3 ± 24.6	111.0 ± 23.5	113.0 ± 25.2	0.381
Total cholesterol, mg/dL	199 ± 36	198 ± 36	199 ± 35	0.777
Triglycerides, mg/dL	93 (66–127)	105 (73–151)	87 (65–116)	<0.001
HDL cholesterol, mmol/L	61 ± 17	52 ± 14	65 ± 17	<0.001
LDL cholesterol, mmol/L	117 ± 30	121 ± 32	115 ± 29	0.023
HbA_1c_, %	5.4 ± 0.3	5.4 ± 0.3	5.3 ± 0.3	0.317
HbA_1c_, mmol/mol	35.0 ± 3.6	35.2 ± 3.4	34.9 ± 3.4	0.317
Fasting glucose, mg/dL	87 ± 11	90 ± 11	86 ± 10	0.002
CGN parameters				
Mean glucose, mg/dL	105 ± 8	106 ± 8	105 ± 8	0.151
SD, mg/dL	13.5 ± 4.4	13.2 ± 4.3	13.7 ± 4.3	0.269
CV, %	14.6 ± 4.3	14.4 ± 4.1	14.7 ± 4.4	0.435
MAGE, mg/dL	27.9 ± 9.4	27.9 ± 10.1	27.9 ± 9.0	0.957

Data are means ± SD, medians [IQR] or n [%]. CGM, continuous glucose monitoring; CV, coefficient of variation; eGFR, estimated glomerular filtration rate; MAGE, mean amplitude of glucose excursion. ^a^For differences between men and women.

As an average, six-years follow-up was performed of non-diabetic patients (interquartile range 4.9–7.3 years). The follow-up period was slightly higher (p=0.021) in women (6.1 (5.0–7.3) years) as compared to men (5.5 (4.5–7.1) years). Of note, 16 women (4.9 %) and 9 men (5.2 %) progressed to diabetes, without significant sex-based differences. Initial HbA_1c_ and fasting glucose concentrations were significantly higher in the subjects who ultimately developed diabetes (5.8 vs. 5.3 %; and 102 vs. 86 mg/dL; p<0.001 in both cases) (Table 2). The patients who experienced progression to diabetes exhibited significantly higher glycemic variability (mean SD 18 vs. 13 mg/dL, p<0.001; CV 17 % vs. 14 %, p=0.004; MAGE 36 vs. 27 mg/dL, p<0.001) and higher mean glycemias (115 vs. 105 mg/dL, p<0.001), as compared to those who did not develop diabetes.

**Table 2:** Markers of glycemic control and glycemic variability in subjects who progressed or not to diabetes.

	Progressed to diabetes type 2		
Variable	No (n=472)	Yes (n=25)	p-Value^a^
HbA_1c_, %	5.3 (0.3)	5.8 (0.4)	<0.001
HbA_1c_, mmol/mol	35 (3)	40 (4)	<0.001
Fasting glucose, mg/dL	86 (10)	102 (12)	<0.001
Mean glucose, mg/dL	105 (8)	115 (9)	<0.001
SD, mg/dL	13.3 (4.2)	18.4 (5.0)	<0.001
CV, %	14.4 (4.2)	16.9 (4.4)	0.004
MAGE, mg/dL	27.4 (9.2)	35.9 (10.3)	<0.001

Data are expressed as means ± SD, or medians [IQR]. CV, coefficient of variation; MAGE, mean amplitude of glucose excursion. ^a^For differences between subjects who developed or not diabetes type 2.

ROC curves were constructed to compare the AUCs (areas under the curve) for the different parameters of glycemic variability, as measured by CGM. This way, we assessed the capacity of these parameters to predict the development of type 2 diabetes and examine the sensitivity and specificity of these variables. According to the ROC analysis, SD had the highest AUC both, for the total population (0.81), men (0.87) and women (0.77) (Table 3). In the non-diabetic population, a cut-off of 14.9 mg/dL for SD yielded a sensitivity of 80 % and a specificity of 72 %. Taking men and women separately, AUCs for SD (0.87 vs. 0.77), CV (0.74 vs.0.62) and MAGE (0.82 vs.0.72) were persistently higher in men. Of the parameters of variability considered, SD showed the highest sensitivity for men (88.9 %), whereas MAGE had the highest specificity for women (78.2 %).

**Table 3:** Sensitivity, specificity and area under the curve for different parameters of glycemic variability to predict diabetes.

Variable	AUC(95 % CI)	p-Value	Cut-off	Sensitivity^a^	Specificity^a^
Total (n=497)					
SD, mg/dL	0.81 (0.73–0.88)	<0.0001	14.9	80.0	72.3
CV	0.67 (0.56–0.77)	0.001	14.7	68.0	59.8
MAGE, mg/dL	0.76 (0.67–0.85)	<0.001	34.2	58.0	81.5
Men (n=173)					
SD, mg/dL	0.87 (0.78–0.96)	<0.001	14.9	88.9	73.0
CV	0.74 (0.61–0.87)	<0.001	14.7	88.7	59.5
MAGE, mg/dL	0.82 (0.70–0.94)	<0.001	28.1	88.8	60.7
Women (n=324)					
SD, mg/dL	0.77 (0.66–0.88)	<0.001	15.4	75.0	74.6
CV	0.62 (0.48–0.76)	0.086	15.5	56.0	66.8
MAGE, mg/dL	0.72 (0.60–0.84)	<0.01	32.3	62.5	78.2

AUC, area under the curve; CV, coeficient of variación; MAGE, mean amplitude of glucose excursion. ^a^Data are expressed as percentages.

## Discussion

This prospective study involving a large sample of non-diabetic subjects representative of the general population assessed the role of several parameters of glycemic variability, as measured by continuous glucose monitoring (CGM), as predictors of progression to type 2 diabetes over a 6-year follow-up period. Although the different metrics considered (SD, CV and MAGE) predict the development of diabetes, SD had the highest AUC on ROC analysis. All AUCs were higher in men than in women for all the parameters studied.

Currently, glycemic variability emerges as a useful tool for evaluating the management of diabetes, as it has demonstrated to be a predictive factor of complications of diabetes. Likewise, elevated glycemic variability makes it difficult to meet the targets of traditional glycemic control parameters, such as HbA_1c_. The amplitude of glycemic variability is known to be positively correlated with the risk of developing all chronic complications of diabetes i.e. neuropathy, retinopathy, chronic kidney disease and macrovascular problems. This association is primarily mediated by an increase in inflammation and oxidative stress, which result from a higher glycemic variability [26], 27]. However, to the best of our knowledge, no prospective studies are available evaluating the role of glycemic variability metrics as measured by CGM as indicators of progression to diabetes in our study populations.

The higher proportion of subjects showing higher glycemic variability who developed diabetes may be partly associated with a higher prevalence of situations in these subjects that favor the development of diabetes. Serum glucose values are homeostatic variables with a high level of instability, even in short periods of time. This higher instability has been suggested [28] to be influenced by different physiological (for example, glucose intake, emotional stress or physical exercise) or pathological (for example, inflammation, infections or endocrine disorders) conditions.

Although current guidelines recommend a CV <36 %, this is applicable to diabetic patients. Therefore, a CV <36 % is associated with stable glycemia [29]. Interestingly, in the non-diabetic population, SD had the highest AUC and a higher sensitivity than CV to predict progression to diabetes. This could be explained by the fact that fluctuations in mean blood glucose levels are much smaller in individuals without diabetes. This study also revealed that MAGE has a lower sensitivity but a higher specificity to identify subjects that will progress to diabetes, many of whom may have irregular dietary habits. It is worth noting that the MAGE index is not useful for assessing the stability of glycemic values or the time in hypoglycemia or hyperglycemia. However, this index is primarily designed to provide information about the degree of glucose level fluctuations between fasting hypoglycemia and posprandial hyperglycemia [30].

In conclusion, this prospective study involving six-year follow-up of around 500 non-diabetic subjects representative of the general population reveals that participants who progressed to type 2 diabetes had higher glycemic variability, as estimated by different CGM metrics (SD, CV and MAGE). These findings open the way for new therapeutic approaches to prevent progression to diabetes.

## References

[1] Akintola AA, Noordam R, Jansen SW, de Craen AJ, Ballieux BE, Cobbaert CM (2015). Accuracy of continuous glucose monitoring measurements in normoglycemic individuals. PLoS ONE.

[2] Rodbard D (2016). Continuous glucose monitoring: a review of successes, challenges, and opportunities. Diabetes Technol Ther.

[3] Bergenstal RM, Ahmann AJ, Bailey T, Beck RW, Bissen J, Buckingham B (2013). Recommendations for standardizing glucose reporting and analysis to optimize clinical decision making in diabetes: the ambulatory glucose profile [AGP]. Diabetes Technol Ther.

[4] Battelino T, Danne T, Bergenstal RM, Amiel SA, Beck R, Biester T (2019). Clinical targets for continuous glucose monitoring data interpretation: recommendations from the international consensus on time in range. Diabetes Care.

[5] Nguyen M, Han J, Spanakis EK, Kovatchev BP, Klonoff DC (2020). A review of continuous glucose monitoring-based composite metrics for glycemic control. Diabetes Technol Ther.

[6] Raj R, Mishra R, Jha N, Joshi V, Correa R, Kern PA (2022). Time in range, as measured by continuous glucose monitor, as a predictor of microvascular complications in type 2 diabetes: a systematic review. BMJ Open Diabetes Res Care.

[7] Taylor PJ, Thompson CH, Brinkworth GD (2018). Effectiveness and acceptability of continuous glucose monitoring for type 2 diabetes management: a narrative review. J Diabetes Investig.

[8] Taylor PJ, Thompson CH, Luscombe-Marsh ND, Wycherley TP, Wittert G, Brinkworth GD (2019). Efficacy of real-time continuous glucose monitoring to improve effects of a prescriptive lifestyle intervention in type 2 diabetes: a pilot study. Diabetes Ther.

[9] Yoo H, An H, Park S, Ryu O, Kim H, Seo J (2008). Use of a real time continuous glucose monitoring system as a motivational device for poorly controlled type 2 diabetes. Diabetes Res Clin Pract.

[10] Cox DJ, Taylor AG, Moncrief M, Diamond A, Yancy WS, Hegde S (2016). Continuous glucose monitoring in the self-management of type 2 diabetes: a paradigm shift. Diabetes Care.

[11] Allen N, Whittemore R, Melkus G (2011). A continuous glucose monitoring and problem-solving intervention to change physical activity and behavior in women with type 2 diabetes: a pilot study. Diabetes Technol Ther.

[12] Allen NA, Fain JA, Braun B, Chipkin SR (2008). Continuous glucose monitoring counseling improves physical activity behaviors of individuals with type 2 diabetes: a randomized clinical trial. Diabetes Res Clin Pract.

[13] Ida S, Kaneko R, Imataka K, Okubo K, Shirakura Y, Azuma K (2020). Effects of Flash Glucose Monitoring on dietary variety, physical activity, and self-care behaviors in patients with diabetes. J Diabetes Res.

[14] Bry L, Chen PC, Sacks DB (2001). Effects of hemoglobin variants and chemically modified derivatives on assays for glycohemoglobin. Clin Chem.

[15] Galindo RJ, Beck RW, Scioscia MF, Umpierrez GE, Tuttle KR (2020). Glycemic monitoring and management in advanced chronic kidney disease. Endocr Rev.

[16] Radin MS (2014). Pitfalls in hemoglobin A1c measurement: when results may be misleading. J Gen Intern Med.

[17] Bergenstal RM, Gal RL, Connor CG, Gubitosi-Klug R, Kruger D, Olson BA (2017). Racial differences in the relationship of glucose concentrations and hemoglobin A1c levels. Ann Intern Med.

[18] Shipman KE, Jawad M, Sullivan KM, Ford C, Gama R (2015). Ethnic/racial determinants of glycemic markers in a UK sample. Acta Diabetol.

[19] Wolffenbuttel BHR, Herman WH, Gross JL, Dharmalingam M, Jiang HH, Hardin DS (2013). Ethnic differences in glycemic markers in patientswith type 2 diabetes. Diabetes Care.

[20] Nielsen LR, Ekbom P, Damm P, Glümer C, Frandsen MM, Jensen DM (2005). HbA_1c_ levels are significantly lower in early and late pregnancy. Diabetes Care.

[21] Rodriguez-Segade S, Rodriguez J, Camiña F, Fernández-Arean M, García-Ciudad V, Pazos-Couselo M (2018). Continuous glucose monitoring is more sensitive than HbA_1c_ and fasting glucose in detecting dysglycaemia in a Spanish population without diabetes. Diabetes Res Clin Pract.

[22] Gude F, Díaz-Vidal P, Rúa-Pérez C, Alonso-Sampedro M, Fernández-Merino C, Rey-García J (2017). Glycemic variability and its association with demographics and lifestyles in a general adult population. J Diabetes Sci Technol.

[23] Aleppo G, Aroda VR, Bannuru RR, Brown FM, Bruemmer D, American Diabetes Association (2023). 2. Classification and diagnosis of diabetes: standards of medical care in diabetes 2023. Diabetes Care.

[24] Hill NR, Oliver NS, Choudhary P, Levy JC, Hindmarsh P, Matthews DR (2011). Normal reference range for mean tissue glucose and glycemic variability derived from continuous glucose monitoring for subjects without diabetes in different ethnic groups. Diabetes Technol Ther.

[25] Hoelzel W, Weykamp C, Jeppsson JO, Miedema K, Barr JR, Goodall I (2004). IFCC reference system for measurement of haemoglobin HbA_1C_ in human blood and the National standardization schemes in the United States, Japan, and Sweden: a method-comparison study. Clin Chem.

[26] Saisho Y (2014). Glycemic variability and oxidative stress: a link between diabetes and cardiovascular disease?. Int J Mol Sci.

[27] Valente T, ArbexVariability AKG (2021). Oxidative stress, and impact on complications related to type 2 diabetes mellitus. Curr Diabetes Rev.

[28] Banday MZ, Sameer AS, Nissar S (2020). Pathophysiology of diabetes: an overview. Avicenna J Med.

[29] Elsayed NA, Aleppo G, Aroda UR, Bannuru RR, Brown FM, Bruemmer D (2023). Glycemic targets: standards of care in diabetes-2023. Diabetes Care.

[30] Sparks JR, Kishman EE, Sarzynski MA, Davis JM, Grandjean PW, Durstine JL (2021). Glycemic variability: importance, relationship with physical activity, and the influence of exercise. Sports Med Health Sci.

